# Development and Validation of an Effective CRISPR/Cas9 Vector for Efficiently Isolating Positive Transformants and Transgene-Free Mutants in a Wide Range of Plant Species

**DOI:** 10.3389/fpls.2018.01533

**Published:** 2018-10-23

**Authors:** Ting Tang, Xiwen Yu, Hong Yang, Qi Gao, Hongtao Ji, Yanxu Wang, Guanbo Yan, Yan Peng, Huifeng Luo, Kede Liu, Xia Li, Chaozhi Ma, Chunying Kang, Cheng Dai

**Affiliations:** ^1^National Key Laboratory of Crop Genetic Improvement, Huazhong Agricultural University, Wuhan, China; ^2^College of Plant Science and Technology, Huazhong Agricultural University, Wuhan, China; ^3^Key Laboratory of Horticultural Plant Biology (Ministry of Education), College of Horticulture and Forestry Sciences, Huazhong Agricultural University, Wuhan, China; ^4^State Key Laboratory of Agricultural Microbiology, College of Plant Science and Technology, Huazhong Agricultural University, Wuhan, China

**Keywords:** CRISPR/Cas9, visual screening, genome editing, *Arabidopsis*, *B. napus*, strawberry, soybean

## Abstract

The CRISPR/Cas9 technique is a highly valuable tool in creating new materials for both basic and applied researches. Previously, we succeeded in effectively generating mutations in *Brassica napus* using an available CRISPR/Cas9 vector *pKSE401*, while isolation of Cas9-free mutants is laborious and inefficient. Here, we inserted a fluorescence tag (sGFP) driven by the constitutive *35S* promoter into *pKSE401* to facilitate a visual screen of mutants. This modified vector was named *pKSE401G* and tested in several dicot plant species, including *Arabidopsis*, *B. napus*, *Fragaria vesca* (strawberry), and *Glycine max* (soybean). Consequently, GFP-positive plants were readily identified through fluorescence screening in all of these species. Among these GFP-positive plants, the average mutation frequency ranged from 20.4 to 52.5% in *Arabidopsis* and *B. napus* with stable transformation, and was 90.0% in strawberry and 75.0% in soybean with transient transformation, indicating that the editing efficiency resembles that of the original vector. Moreover, transgene-free mutants were sufficiently identified in *Arabidopsis* in the T2 generation and *B. napus* in the T1 generation based on the absence of GFP fluorescence, and these mutants were stably transmissible to next generation without newly induced mutations. Collectively, *pKSE401G* provides us an effective tool to readily identify positive primary transformants and transgene-free mutants in later generations in a wide range of dicot plant species.

## Introduction

The CRISPR/Cas9 mediated genome-editing technology provides unprecedented tools to precisely edit DNA sequences in animals and plants (Mali et al., 2013; Gao, 2018). This technology requires expression of the Cas9 protein, production of a guide RNA (gRNA) that complements the target DNA sequences, and the existence of an NGG protospacer adjacent motif (PAM) site in the target sequence (Cong et al., 2013; Mali et al., 2013). Briefly, genome editing mediated by CRISPR/Cas9 utilizes a 20-bp gRNA that directs the Cas9 nuclease to the target site by base pairing. Cas9 cuts the target site to generate a double strand break (DSB). Mutations are introduced during the DNA repairing process. Because of its simplicity, CRISPR/Cas9 has been widely adopted. Several CRISPR vectors have been developed for genome editing in plants (Cong et al., 2013; Feng et al., 2014; Li et al., 2014; Ma et al., 2015; Xie et al., 2015; Gao et al., 2016; Pan et al., 2016; Ding et al., 2018). It was proven that CRISPR/Cas9-mediated genome editing technology could successfully generate various heritable mutations in plant species (Bortesi et al., 2016; Yang et al., 2017).

After transformation, the first mission is to isolate primary transformants with expected mutations. To this end, restriction enzyme digestion or sequencing of the PCR amplicons was usually performed (Bortesi et al., 2016; Pan et al., 2016; Yang et al., 2017). However, both methods are time- and money-consuming and laborious. Next, mutants without the T-DNA insertion may need to be identified, which are favored in both basic and applied researches because of the following reasons. First, prolonged existence of CRISPR/Cas9 in the mutants would greatly increase the risk of producing off-target mutations. Second, transgene-free materials are more easily accepted by the public. Cas9-free mutants could be obtained by self-crossing or backcrossing. In *Brassica napus*, for instance, by using gene specific primers (such as *Cas9*), 10.9% (58/530) of the mutant plants in the T1 generation lost the Cas9 transgene by self-crossing (Yang et al., 2017), a ratio lower than what was expected, which need larger populations to get Cas9-free mutant. Therefore, an easy method to screen for the mutants in need in both primary transformants and their offspring is highly required.

As previously reported, making use of fluorescent proteins as visible markers would not only speed up the screening of positive transformants but also help to isolate transgene-free offspring (Gao et al., 2016; Hu et al., 2017). In this study, we developed a new vector used for visual screen of transgenic plants by adding a *35S::sGFP* cassette in the CRISPR/Cas9 vector *pKSE401*. This modified vector was named *pKSE401G* and successfully applied in *B. napus*, *Arabidopsis*, strawberry, and soybean, respectively. The mutation frequency of each target gene was as high as that generated by the original vector (Yang et al., 2017). Moreover, Cas9-free mutants could be readily isolated according to the absence of GFP fluorescence, and the mutations were stably inherited into the next generation. Our strategy of using GFP and dual gRNAs provides an efficient tool to screen for edited plants and isolate Cas9-free mutants with the desired mutations.

## Results

### Generation of a New CRISPR/Cas9 Vector Used for Visual Screen of Transformants

**FIGURE 1:**
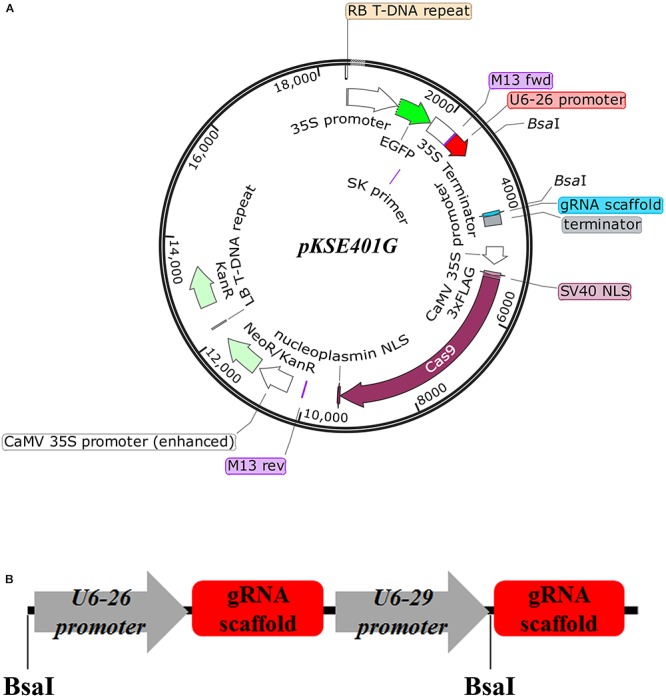
Design of the vector *pKSE401G*. **(A)** Schematic representation of the *pKSE401G* vector that contains a Cas9 and GFP expression cassette driven by the *35S promoter* and a *U6* promoter-controlled gRNA production unit. NLS, nuclear localization signal. *BsaI*, *BsaI* cutting sites. **(B)** The fragment that would be inserted into *pKSE401G* to make construct. *U6-26p* and *U6-29p* are two *Arabidopsis U6* promoters that drive the two sgRNAs, respectively. gRNA-Sc: gRNA scaffold.

Previously, an existing CRISPR/Cas9 vector *pKSE401* was successfully applied into *Arabidopsis* and *B. napus* to generate mutants with a high efficiency (Xing et al., 2014; Yang et al., 2017). However, verification of positive transformants and isolation of transgene-free offspring are tedious. To overcome this drawback, an optimized CRISPR/Cas9 vector, named *pKSE401G*, was made by inserting a *35S::sGFP* cassette into *pKSE401* (Figure 1A) (Xing et al., 2014). Other parts of the vector remained intact. Specifically, *zCAS9-NLS* was under the control of two constitutive 35S promoters, and expression of the two sgRNA scaffolds was driven by the *Arabidopsis U6-26* and *U6-29* promoter, respectively (Figure 1B) (Xing et al., 2014). To make constructs, two sgRNAs should be designed for one target gene. The fragments could be amplified by over-lapping PCR using the vector *pCBC-DT1T2* (Xing et al., 2014) as template, and then inserted into *pKSE401G* that was linearized by the type II restriction enzyme *BsaI* through the Golden Gate Assembly method.

### Mutation of *RPK1* in *Arabidopsis*

**FIGURE 2:**
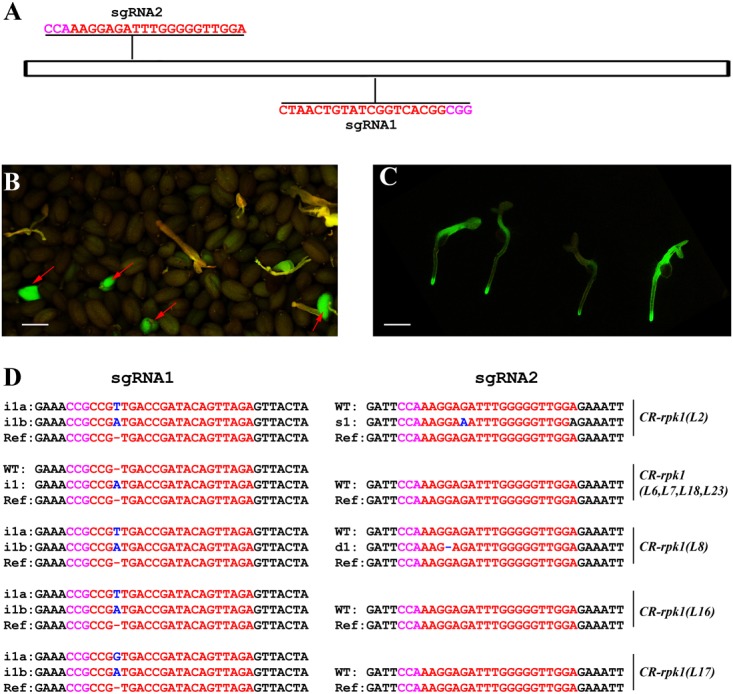
Generation of *CR-rpk1* mutants in *Arabidopsis*. **(A)** The target sites of sgRNA1 and sgRNA2 in *RPK1*. The PAM sites (NGG or CCN) are indicated with magenta. **(B,C)** Visual screen of positive transgenic *Arabidopsis* in the T1 generation. GFP signal was visualized in seeds **(B)** and seedlings **(C)**. **(D)** Mutations induced at the sgRNA1 and sgRNA2 target sites in *RPK1* in different lines in the T1 generation. The PAM sequence is indicated with magenta. The sgRNA is indicated with red. The mutation sites are indicated with blue. i, insertion; d, deletion; s, substitution. d#, # of base pair (bp) deleted from target site; i#, # of bp inserted at target site; i#a and i#b, different nucleotide insertion.

In order to assess the editing efficiency of *pKS401G*, two sgRNAs were first designed for mutating the *Arabidopsis* gene *RECEPTOR-LIKE PROTEIN KINASE* 1(*RPK1*) (Figure 2A). This gene encodes a leucine rich repeat receptor like kinase that positively regulates abscisic acid (ABA) signaling transduction in *Arabidopsis* (Osakabe et al., 2005). The construct was transformed into wild-type *Arabidopsis Col-0.* In the T1 generation, some green seeds were easily identified under the fluorescence microscope, which are potential positive primary transformants harboring the GFP-expressing cassette (Figure 2B). When these green seeds germinated, the green fluorescence signal was still present in the young seedlings (Figure 2C). These *CR-rpk1* (*CRISPR-rpk1*) plants were further confirmed by PCR based genotyping using the gene specific primers of *Cas9* (Supplementary Figure S1A). As a result, all the GFP-positive lines contained the Cas9 sequence.

Next, the mutation frequency was analyzed by direct sequencing in the T1 generation. All sequencing results of each amplicon derived from individual transformants were decoded by using the website DSDecode^1^ (Liu et al., 2015). The results showed that both target sites were successfully edited (Figure 2D), however, the editing efficiencies were significantly different. At sgRNA1 target site, 33.3% (9 of 27) of the T1 plants contained detectable mutations. In contrast, the mutation rate was much lower (7.4%, 2 of 27) at sgRNA2 target site. A low proportion of homozygous mutations (3.7%, 1 of 27) could also be identified.

**FIGURE 3:**
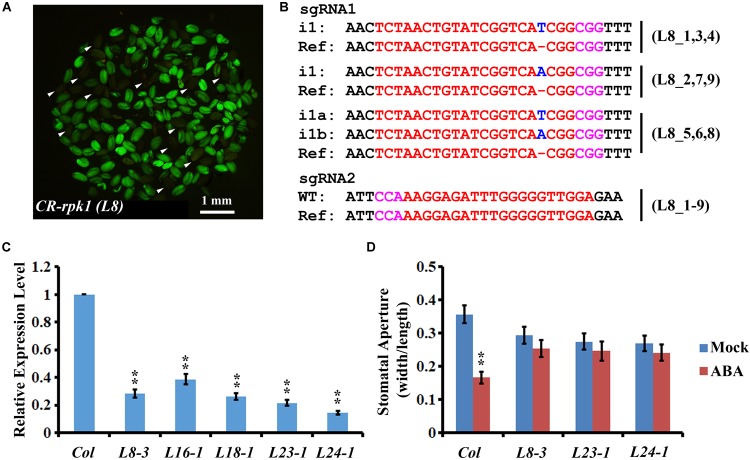
Visual screen and characterization of Cas9-free *CR-rpk1* plants in the T2 generation. **(A)** Visual screen of potential Cas9-free *CR-rpk1* seeds indicated by the white arrows in the T2 generation. **(B)** Mutations induced at the sgRNA1 target site in *CR-rpk1* in the L8 plants in the T2 generation. The PAM sequence is indicated with magenta. The sgRNA is indicated with red. The mutation sites are indicated with blue. i#, # of bp inserted at target site; i#a and i#b, different nucleotide insertion. All of the sgRNA2 target sites are wild type. **(C)** Expression level of *RPK1* in *CR-rpk1*mutants obtained by qRT-PCR. *ACTIN* was used as the internal control. Data are means ± SD obtained from three biological replicates. ^∗∗^*P* < 0.01, Student’s *t*-test. **(D)** Stomatal aperture in wild type and three *CR-rpk1* mutants in response to ABA treatment for 1 h. Data are means ± SD (*n* = 50). ^∗∗^*P* < 0.01, Student’s *t*-test.

Cas9-free mutants provide reliable materials for gene functional studies and crop improvement. To isolate Cas9-free mutants, the seeds from nine T1 transgenic lines were harvested for screening. The seeds that did not contain the CRISPR/Cas9 insertion were selected based on the absence of GFP fluorescence (Figure 3A). In total, 251 seedlings were screened. GFP signal was detected in 82.1% (206/251) of transgenic plants, while only 17.3% (45/251) of plants did not have the GFP signal (Supplementary Table S1). As lack of green fluorescence could be due to gene silencing (Gao et al., 2016), the GFP-negative plants were further checked by PCR using *Cas9-, GFP-, and NPTII*-specific primers, respectively (Supplementary Figure S2, left panel). Consequently, 37 plants were confirmed as Cas9-free. Then genotyping of these 37 Cas9-free plants revealed that 56.8% (21/37) were homozygous mutants, and 21.6% (8/37) were bi-allelic mutants. Thus, a total of 78.4% had defects in both alleles (Figure 3B and Supplementary Table S2). Among all the homozygotes and bi-allelic mutants, 100.0% (29/29) of them carried a 1-bp insertion (i1) (Supplementary Table S2). The frequency of heterozygotes was 16.2% (6/37), and 5.4% (2/37) were not edited (Supplementary Table S2). The results demonstrated that screening Cas9-free material is more efficient owing to application of the GFP reporter.

To determine whether the mutations in the Cas9-free plants could be stably transmitted to offspring, 20 individual plants obtained from Cas9-free lines L8-3 and L18-1 in T2 were genotyped. All detected plants were homozygous at sgRNA1 target site with the same mutations as their parents, and no new mutation was identified at sgRNA2 target site (Supplementary Table S3). These results suggested that the mutations in Cas9-free plants could be stably transmitted to the next generation following a Mendelian law in *Arabidopsis*.

Next, we examined the expression level of *RPK1* in the *CR-rpk1* mutants. Several independent lines were examined, in all of which the transcript level was greatly reduced (Figure 3C and Supplementary Figure S4A), probably caused by non-sense mediated decay. As previously reported, one important phenotype of the *RPK1* mutant is less sensitive to ABA in *Arabidopsis* (Osakabe et al., 2005). Therefore, we measured the stomatal aperture after ABA treatment for 1 h in three independent *CR-rpk1* mutants. As expected, the stomatal aperture became much smaller in wild type after ABA treatment; in contrast, stomatal closure was largely inhibited in the *CR-rpk1* mutants (Figure 3D). These data demonstrated that the *pKSE401G* vector could be used for gene functional studies in *Arabidopsis*.

### Mutation of *BnaARF2* With Four Copies in *B. napus*

**FIGURE 4:**
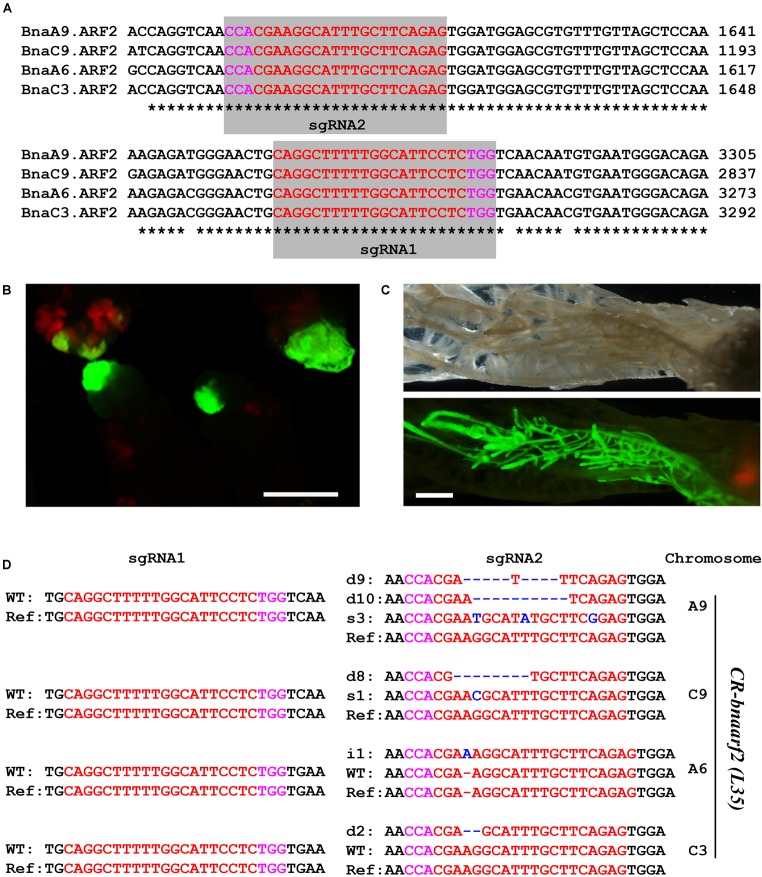
Generation of *CR-bnaarf2* mutants in *B. napus*. **(A)** Partial coding sequence alignment of the four *BnaARF2*s containing the two sgRNA target sites. The PAM sequences are indicated with magenta. The sgRNA is indicated with red. **(B,C)** Visual screen of positive transgenic calli **(B)** and roots **(C)**. **(D)** Mutations induced at the sgRNA1 and sgRNA2 target sites in four *BnaARF2s* in L35 in the T0 generation. The PAM sequence is indicated with magenta. The sgRNA is indicated with red. The mutation sites are indicated with blue. i, insertion; d, deletion; s, substitution. d#, # of base pair (bp) deleted from target site; i#, # of bp inserted at target site; i#a and i#b, different nucleotide insertion.

We then tested genome editing efficiency of *pKSE401G* in the allotetraploid plants *B. napus*. *BnaARF2* was selected as the target gene, which is the ortholog of *Arabidopsis AUXIN RESPONSE FACTOR 2* (*ARF2*) gene, a repressor in auxin signaling (Schruff et al., 2006). There are four *BnaARF2* paralogous genes in the *B. napus* genome, namely, *BnaA6.ARF2*, *BnaA9.ARF2*, *BnaC3.ARF2*, and *BnaC9.ARF2* (Supplementary Figure S5). Two sgRNAs were designed to target the conserved sequences among the four paralogous genes (Figure 4A). GFP signal was tracked as a selection marker of positive calli and regenerated plants during the transformation process (Figures 4B,C), which greatly increased work efficiency. Finally, a total of five individual GFP-positive plants were isolated. All of these transformants were further confirmed by PCR using *Cas9*-specific primers (Supplementary Figure S1B).

Next, we used Sanger sequencing to assess the editing efficiency. Each of the four *BnaARF2* genes was, respectively, examined in the five transgenic plants. As a result, the mutation frequency at sgRNA1 target site is always 20% for each of the four genes, while the mutation frequency at sgRNA2 target site ranged from 60.0 to 100.0% (Table 1). With a combination of the two target sites, three transgenic plants (L34, L35, and L37) were quadruple mutants, one (L38) was triple mutant (*BnaA9.ARF2 BnaC9.ARF2*, and *BnaC3.ARF2*), and one (L39) was double mutant (*BnaC9.ARF2* and *BnaC3.ARF2*). Among all of the types of mutations, 47.2% (17/36) were deletions, 25.0% (9/36) were insertions, 13.9% (5/36) were combined mutations, and 13.9% (5/36) were substitutions (Figure 4D and Supplementary Figure S3). The number of deleted nucleotides ranged from 1 to 10 (Supplementary Figure S3). The average mutation frequency in the T0 generation in *B. napus* was much higher than that in the T1 generation in *Arabidopsis* (52.5% vs. 20.4%).

**Table 1:** Percentage of mutated plants in the T0 generation in *Brassica napus.*

		T0 generation			Homozygous Mutations in T1(T0)		Number of plants with GFP signal	Number of plants with Cas9	% of plants mutated at different target genes
					
Target gene	sgRNA	Number of Plants examined	Number of plants with mutations	Mutation rate (%)	Number	Rate (%)			
*BnaA6.ARF2*	sgRNA1	5	1	20.0	0	0	5	5	One gene mutated: 0
	sgRNA2	5	3	60.0	0	0			
*BnaA9.ARF2*	sgRNA1	5	1	20.0	0	0	5	5	Two genes mutated: 20.0
	sgRNA2	5	5	100.0	0	0			
*BnaC3.ARF2*	sgRNA1	5	1	20.0	0	0	5	5	Three genes mutated: 20.0
	sgRNA2	5	5	100.0	0	0			
*BnaC9.ARF2*	sgRNA1	5	1	20.0	1	20.0	5	5	Four genes mutated:60.0
	sgRNA2	5	4	80.0	0	0			
Total (average)		5		(52.5)		(2.5)	5	5	

**FIGURE 5:**
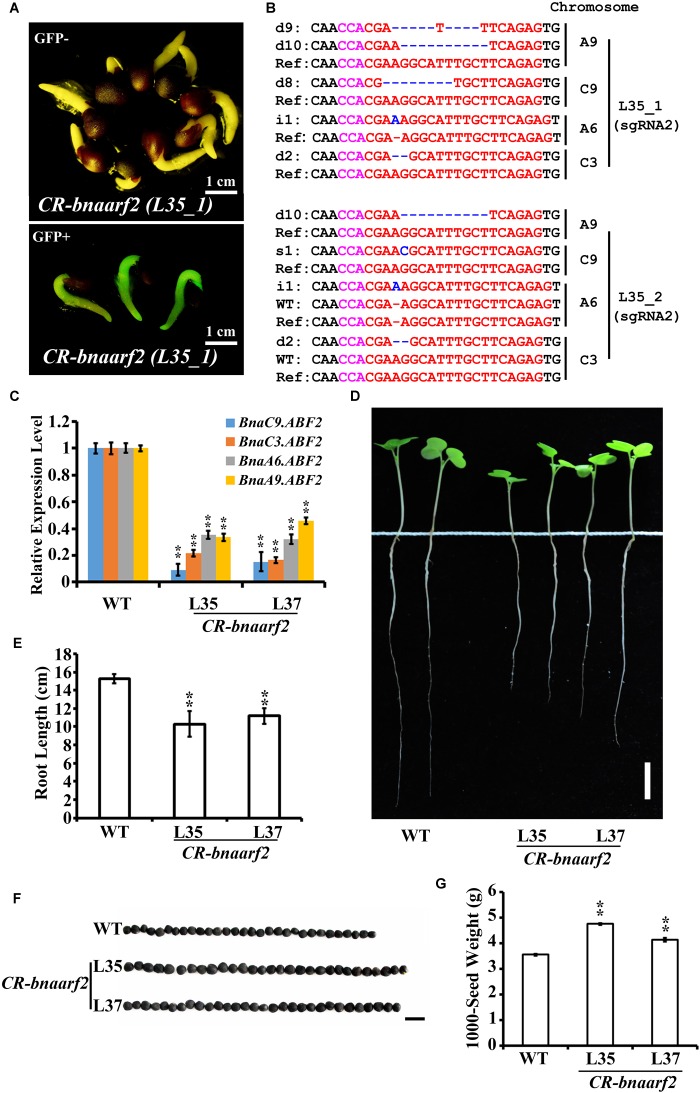
Visual screen and characterization of Cas9-free *CR-bnaarf2* mutants in the T1 generation. **(A)** Visual screen of potential Cas9-free *CR-bnaarf2* (L35) based on GFP signal. GFP signal was visualized in roots. **(B)** Mutations induced at the sgRNA1 target site in *CR-bnaarf2* (L35) plants in the T1 generation. The PAM sequence is indicated with magenta. The sgRNA is indicated with red. The mutation sites are indicated with blue. i, insertion; d, deletion; s, substitution. d#, # of base pair (bp) deleted from target site; i#, # of bp inserted at target site, i#a and i#b, different nucleotide insertion. **(C)** Expression level of *BnaARF2* paralogous genes in *CR-bnaarf2* mutants obtained by qRT-PCR. *BnaACTIN* was used as the internal control. Data are means ± SD obtained from three biological replicates. **(D)** Image showing the root morphology of *CR-bnaarf2* quadruple mutants. Bar = 2 cm. **(E)** Bar graph showing the root length of *CR-bnaarf2* quadruple mutants. Data are means ± SD (*n* = 10). **(F)** Image showing the seed size of *CR-bnaarf2* quadruple mutants. Bar = 0.5 cm. **(G)** Bar graph showing the 1000-seed weight of *CR-bnaarf2* quadruple mutants. SD values were calculated from three individual experiments. *n* = 10 of each plant per experiment. In C-G, L35 and L37: two individual T1 transgenic lines. WT: *Westar*. In **(C,E,G)**, ^∗∗^*P* < 0.01, Student’s *t*-test.

The five positive transgenic plants were self-crossed to obtain the T1 population. In T1, GFP signal was examined in a total of 95 seedlings from L35, L37, L38, and L39 (Figure 5A). Consequently, 24 seedlings (25.7%) lost the GFP signal (Supplementary Table S1). All of these GFP-negative plants were further confirmed by using *Cas9*-*, GFP-* and *NPTII*-specific primers, respectively (Supplementary Figure S2, right panel). Next, mutations in *BnaARF2s* at the sgRNA2 target site were examined in 20 Cas9-free plants in T1 from L35, L37, and L38 (Figure 5B, Table 2, and Supplementary Table S4). Homozygous mutations were identified in *BnaC9.ARF2*, *BnaA6.ARF2*, and *BnaC3.ARF2* (Supplementary Table S4). Bi-allelic mutations were identified in *BnaA9.ARF2* and *BnaC9.ARF2* (Supplementary Table S4). Heterozygotes were found in *BnaA6.ARF2*, *BnaA9.ARF2*, and *BnaC3.ARF2* (Supplementary Table S4). The results suggested that fluorescence screening would speed up getting Cas9-free mutants in *B. napus*.

**Table 2:** Percentage of mutated sites in Cas9-free *B. napus* plants in the T1 and T2 generations.

		T1 generation			T2 generation			GFP signal	Cas9
Target gene	sgRNA	Number of Plants examined	Number of plants with mutations	Mutation rate (%)	Number of Plants examined	Number of plants with mutations	Mutation rate (%)		
*BnaA6.ARF2*	sgRNA1	10	0	0	10	0	0	N	N
	sgRNA2	10	10	100.0	10	10	100.0		
*BnaA9.ARF2*	sgRNA1	13	0	0	10	0	0	N	N
	sgRNA2	13	13	100.0	10	10	100.0		
*BnaC3.ARF2*	sgRNA1	20	0	0	10	0	0	N	N
	sgRNA2	20	20	100.0	10	10	100.0		
*BnaC9.ARF2*	sgRNA1	17	0	0	10	0	0	N	N
	sgRNA2	17	17	100.0	10	10	100.0		
Average		20	(15)	(50.0)	10	10	(50.0)		

To test whether the mutations in the Cas9-free plants could be stably transmitted to the next generation in *B. napus*, we compared the mutations between one T1 plant with its 10 offspring in T2 (*L35-1*). As shown in Supplementary Table S5, the mutations in the four *BnaARF2* genes were either homozygous or bi-allelic in the one T1 plant at sgRNA2 target sites, and these mutations remained the same in the 10 T2 plants. In addition, there is no newly induced mutation at sgRNA1 target site. These results indicated that the mutations in Cas9-free mutants were inheritable following a Mendelian law in *B. napus*.

Among the transgenic plants, the quadruple mutants of *BnaARF2s*, called *CR-bnaarf2*, were identified in L35 and L37, respectively. Furthermore, all of the four *BnaARF2* genes were remarkably down-regulated in these mutants compared to wild type (Figure 5C and Supplementary Figure S4B). In *Arabidopsis*, *arf2* mutants are defective in several aspects, such as reduced fertility, delayed senescence, enlarged rosette leaves, seeds and cotyledons (Schruff et al., 2006). We also examined the phenotypes of *CR-bnaarf2*. The primary roots were much shorter than WT (Figures 5D,E), the seed size and 1000-seed weight were significantly increased compared to WT (Figures 5F,G), indicating that *BnaARF2* is a positive regulator of root elongation and a negative regulator of seed enlargement in *B. napus*. Together, these data suggested that the *pKSE401G* vector performed well in targeted genome editing in *B. napus*.

### Mutation of *FveMYB10* in Fruit of *Fragaria vesca* by Transient Transformation

**FIGURE 6:**
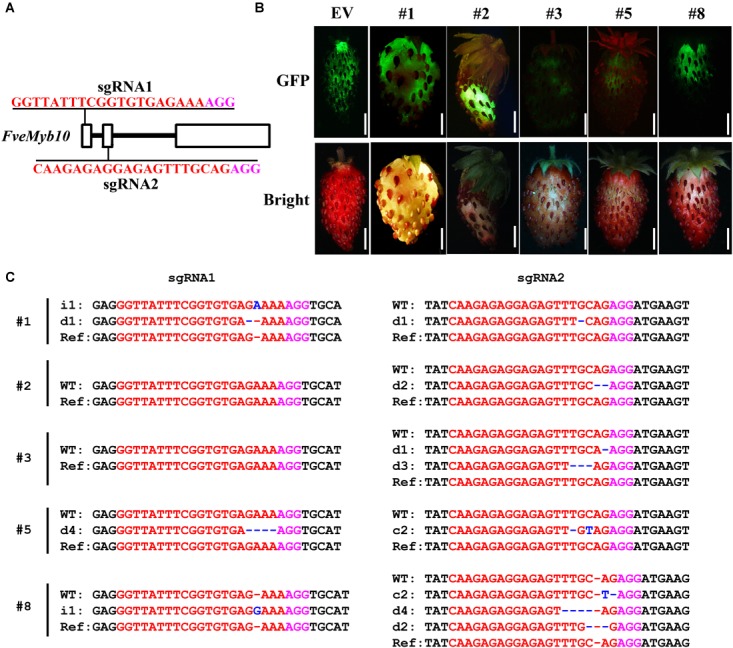
Phenotype and genotype of strawberry fruit transiently transformed with *CR-FveMYB10.* **(A)** The target sites of sgRNA1 and sgRNA2 in *FveMyb10*. **(B)** Fruit transiently expressing *CR-FveMYB10* by agro-infiltration. GFP signal was shown in upper panel, and the same fruit was shown in lower panel. EV, empty vector. Bar = 0.5 cm. **(C)** Mutations induced at the sgRNA1 and sgRNA2 target sites in individual fruit. The mutation sites are indicated with blue. i, insertion; d, deletion; c, combined. d#, # of base pair (bp) deleted from target site; i#, # of bp inserted at target site, c#, combined mutations. In **(A,C)**, the PAM sequence is indicated with magenta; the sgRNA is indicated with red.

The *U6* and *U3* promoters used for driving the two sgRNAs have a major influence on genome editing efficiency (Bortesi et al., 2016; Pan et al., 2016; Yang et al., 2017). *Arabidopsis U6* and *U3* promoters worked well in *B. napus*, perhaps because the two species are closely related. Then we would like to test the genome editing efficiency of *pKSE401G* in other dicot plant species which is evolutionarily far from *Arabidopsis*. First, we tried it in the diploid woodland strawberry *Fragaria vesca* in *Rosaceae* family. The well-studied gene *FveMyb10* was chosen as the target, as transient knock-down of *FveMyb10* provoked loss of coloration in fruit receptacle (Lin-Wang et al., 2014). Similarly, two sgRNAs were designed for targeting *FveMyb10* (Figure 6A). Ten individual fruits were injected, and the GFP signal was observed 7 days after injection (Figure 6B, upper panel). Compared to control fruit (Figure 6B, *EV*), less anthocyanin was accumulated in the injected fruit (Figure 6B, down panel). Moreover, mutations could be identified in *FveMyb10* at both the sgRNA1 and sgRNA2 target sites in all the injected fruit (Figure 6C and Supplementary Figure S6). These results indicated that *pKSE401G* is effective in strawberry by transient transformation.

### Mutation of *GmNFR1a* in the Roots of Soybean by Transient Transformation

**FIGURE 7:**
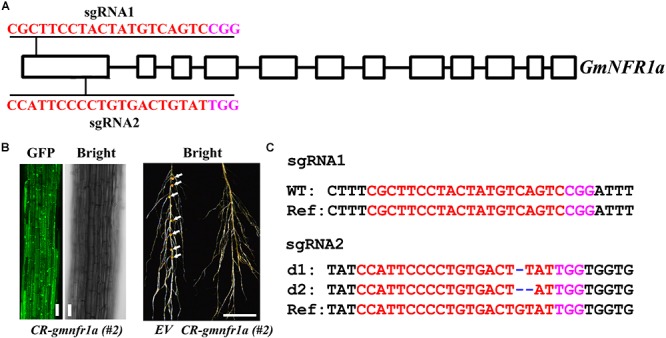
Phenotype and genotype of soybean roots transiently transformed with *CR-GmNFR1a*. **(A)** The target sites of *sgRNA1* and *sgRNA2* in *GmNFR1a*. **(B)** Root transiently expressing *CR-GmNFR1a* by agro-transformation. Root with GFP signal was shown in the left, and root with induced nodules was shown in the right. EV, empty vector. Bar = 10 μm (left); Bar = 1cm (right). White arrows indicated the nodules. **(C)** Mutations induced at the sgRNA1 and sgRNA2 target sites in *GmNFR1a* in root #2. d#, # of base pair (bp) deleted from target site. In **(A,C)**, the PAM sequence is indicated with magenta; the sgRNA is indicated with red.

We further assessed the genome editing efficiency of *pKSE401G* in soybean. Two sgRNAs were designed for *GmNFR1a* (Nod Factor Receptor 1a) (Figure 7A), encoding a LysM receptor-like kinase required for nodulation in the roots of legumes (Indrasumunar et al., 2011). The GFP fluorescence was visualized in the roots after being transfected by agrobacteria (Figure 7B, left panel). In contrast to wild type, the nodules were completely inhibited (Figure 7B, right panel and Supplementary Figure S7). The genotyping result showed that *GmNFR1a* was mutated at both the sgRNA1 and sgRNA2 target sites (Figure 7C and Supplementary Figure S8). The results in strawberry and soybean indicated that *pKSE401G* could sufficiently generate mutations in a wide range of plant species.

### No Off-Targets Are Discovered in Transgenic Plants

Low-frequency cases of off-target cleavage have been reported for CRISPR/Cas9 in plant research (Zhang et al., 2014; Pan et al., 2016). To detect the off-target events, potential off-target loci that are highly homologous to sgRNAs of *RPK1* and *BnaARF2* were predicted by the online tool CRISPR-P^2^ (Lei et al., 2014). Previous reports revealed that the 12 nucleotides adjoining the PAM, designated as “seed sequence,” are critical for recognition specificity and cleavage efficiency of Cas9 (Xie and Yang, 2013; Zhang et al., 2014; Yan M. et al., 2015). The off-target sequences usually bear 1–3 mismatches in the ‘seed sequence.’ At least three of the most likely off-target sites for each sgRNA were examined in total 50 randomly selected plants by using gene specific primers (Supplementary Table S6). No mutations were found in the putative off-target sites (Table 3), indicating that sequence editing induced by the usage of *pKSE401G* is highly specific.

**Table 3:** Detection of mutations at the putative off-target sites in *Arabidopsis* and *B. napus.*

Target	Putative off-target sites	Putative off-target locus	Putative off-target sequence	Number of mismatch bases	Number of plants examined	Number of mutations
RPK1-sgRNA2	OFF1	1: -7095034	TTCAATCCCCAAAAATCCTTCGG	4	50	0
	OFF2	1: -17399753	TTCAACCCACAAAGATCCTTTAG	4	50	0
BnaARF2-sgRNA1	OFF1	chrC02: +23259650	TCGTATTTTTGGCATTCCTCTGG	4	50	0
	OFF2	chrC05: -42330573	CAGTCTTGGTGCCATTCCTCTGG	4	50	0
	OFF3	chrA08: -17351862	CAGACTTCTTCGGATTCCTCTGG	4	50	0
BnaARF2-sgRNA2	OFF1	chrC02:-39398407	CTCTGAAGCGAATGGCTTCGTGG	2	50	0
	OFF2	chrA03_random: +3859941	CACTGAAGGAATTGCCTTCGAGG	3	50	0
	OFF3	chrC06: +18007104	CTCTGAAGCAAATGACTTTGTGG	2	50	0

*The PAM motif (NGG) is indicated with magenta; mismatched bases are indicated with red*.

## Discussion

The CRISPR/Cas9 technique is highly efficient in genome editing, which provides mutants for gene function studies and crop improvement (Gao, 2018). In this work, we designed a new CRISPR/Cas9 vector that efficiently generated mutations in *Arabidopsis*, *B. napus*, *F. vesca*, and *Glycine max*. The editing events were closely associated with the presence of GFP signal in the examined plants. Based on fluorescence signal screening, the mutant candidates would be readily isolated from a large population, which helps to save time and labor. The mutation frequency ranged from 33.3 to 100%, which is comparable to the results in other plant species as well as in *B. napus* using the original vector *pKSE401* (Yang et al., 2017). The mutations were stably inherited from T1 to T3 in *Arabidopsis* and from T0 to T2 in *B. napus*. More importantly, this vector induced mutations not only in one target gene but also in up to four homologous genes without any reduction of efficiency. Collectively, this new vector is able to efficiently create ideal materials for functional studies.

Prolonged existence of the CRISPR/Cas9 cassette in the mutants greatly increases the risk of producing off-target mutations (Gao et al., 2016). Through self-crossing or backcrossing, Cas9-free mutants could be generated in the T2 generation (Zhang et al., 2014; Pan et al., 2016; Yang et al., 2017). Cas9-free mutants could also be created by delivering the Cas9-sgRNA ribonucleoprotein complex or the CRISPR-Cas9 DNA/RNA into plant cells through particle bombardment or protoplast transfection (Woo et al., 2015; Zhang Y. et al., 2016). However, both methods have shortcomings. Self-crossing or backcrossing is laborious and time-consuming, while the second way is difficult to be achieved within crop plants and needs specialized equipment (e.g., gene gun) or expensive consumables such as gold particles. Moreover, regeneration of plants from the protoplast of *Brassicas* is genotype dependent (Woo et al., 2015). Fluorescence protein is a visible reporter for transgenic material screening. Based on fluorescence “on” or “off,” potential Cas9-free plants were identified sufficiently (Gao et al., 2016). However, occasionally absence of GFP signal might be caused by gene silencing, that is, the T-DNA insertion is still present in the genome (Gao et al., 2016). Therefore, fluorescence signal screening should be combined with PCR amplification using transgene specific primers to ensure loss of the T-DNA insertion in the genome.

Recent data suggested that the mutation frequency could be greatly increased by using tissue-specific or other endogenous promoters with a high activity to drive Cas9 expression than using constitutive promoters, such as the *CaMV 35S promoter* (Yan L. et al., 2015; Zhang K. et al., 2016; Zhang et al., 2017). The expression of sgRNA also affects the editing efficiency. For example, mutation frequencies could be increased two–sevenfold when the intrinsic *U6* promoter is used to drive sgRNA expression in soybean and liverwort, rather than the *Arabidopsis U6* promoter (Sugano et al., 2014; Du et al., 2016). However, high mutation frequencies were observed in *B. napus*, strawberry and soybean when using the *Arabidopsis U6* promoter, suggesting that *Arabidopsis U6* promoter is also suitable for genome editing in other dicot plants. In this study, the transgenic plants with no or low level expression of *Cas9* were eliminated by GFP screening, which may increase the ratio of edited plants. We also noticed that the editing frequency is much lower in *Arabidopsis* T1 generation than that in *B. napus* T0 generation. This lower editing efficiency in *Arabidopsis*, which is transformed via the floral dip method, may be due to the relatively low expression levels of *Cas9* in the embryo sac when driven by the *35S* promoters (Yan L. et al., 2015; Zhang K. et al., 2016; Zhang et al., 2017). Embryo-specific promoters (such as *YAO* promoter) may increase the editing frequency in *Arabidopsis* by increasing the expression level of *Cas9* and sgRNAs during plant reproduction (Yan L. et al., 2015; Zhang K. et al., 2016; Zhang et al., 2017).

Apart from the expression levels of *Cas9* and *sgRNAs*, the CRISPR/Cas9-induced editing efficiency in plants may be affected by the sequence composition (such as GC content) of targets and the secondary structure of the target-sgRNAs (Ma et al., 2015). Previous studies indicated that higher GC content of sgRNAs is usually associated with higher mutation frequencies (Doench et al., 2014; Ma et al., 2015). In this study, the six sgRNAs targeting *RPK1*, *BnaARF2*, and *GmNFR1a* had the same GC content (50%), but their mutation rates were quite different (Supplementary Table S7). This suggested that certain features, apart from GC content, in sgRNAs greatly affected editing efficiency. By comparing non-functional and functional sgRNAs, researchers found that the 3′ end of sgRNAs, also known as the “seed region,” plays a more important role in determining the recognition efficiency (Xie and Yang, 2013; Zhang et al., 2014; Yan M. et al., 2015). In addition, base accessibility of guide sequence at positions 51–53 was generally associated with accessibility of the end of the seed region (Wong et al., 2015).

## Materials and Methods

### Plant Materials and Growth Condition

The *B. napus* variety *Westar*; *Arabidopsis* variety *Col*; the strawberry variety Ruegen (Ru F7-4, red-fruited) (Slovin et al., 2009), and the soybean variety Williams 82 (W82) were used as wild-types in this study. The plants were cultivated in a growth room under a light intensity of 100 μmol m^-2^ s^-1^ with a 16/8 h light/dark photoperiod at 22°C (*B. napus*, *Arabidopsis* and strawberry) or 25°C (soybean).

### Plant Transformation

For *Arabidopsis* transformation, the CRISPR/Cas9 constructs were transformed into *Arabidopsis* wild-type Col-0 through floral dipping (Clough and Bent, 1998). The procedure of *Agrobacterium*-mediated *B. napus* transformation was carried out as previously described (Zhang et al., 2015). Briefly, the explants were incubated in the *Agrobacterium*-infection buffer (MS 4.43 g L^-1^; sucrose 30 g L^-1^; acetosyringone 100 mM L^-1^; pH 5.8–5.9) for 20 min, then transferred to M1 medium plates (MS 4.43 g L^-1^; sucrose 30 g L^-1^; acetosyringone 100 mM L^-1^; mannitol 18 g L^-1^; 2,4-D 1 mg L^-1^; kinetin 0.3 mg ml^-1^; pH 5.8–5.9) and kept in dark for 48 h. Afterwards, the explants were transferred to M2 medium plates (MS 4.43 g L^-1^; sucrose 30 g L^-1^; acetosyringone 100 mM L^-1^; mannitol 18 g L^-1^; AgNO_3_ 4 mg L^-1^; 2,4-D 1 mg L^-1^; kinetin 0.3 mg mL^-1^; Timentin 270 mg L^-1^; pH 5.8–5.9) with proper selection antibiotics to induce callus growth. The calli were transferred to M3 (MS 4.43 g L^-1^; glucose 10 g L^-1^; xylose 0.25 g L^-1^; zeatin 2 mg L^-1^; IAA 0.1 mg L^-1^; Timentin 270 mg L^-1^; pH5.8-5.9) and followed by M4 (MS 2.22 g L^-1^; sucrose 10 g L^-1^; IBA 0.5 mg L^-1^; Timentin 135 mg L^-1^; pH 5.8–5.9) medium to let shoot and root regenerate, respectively.

The strawberry fruit transient transformation assay was performed as described (Luo et al., 2018). Briefly, a single agrobacterium (GV3101) colony was picked and grown in 2 ml of liquid LB medium until OD_600_ reached around 0.8 to 1.0. Then the culture was spun down and resuspended in the infiltration buffer (MS 4.43 g L^-1^; sucrose 20 g L^-1^) to reach an OD_600_ of 0.8. Fruit at the white stage were used for injection. The color phenotype was examined 1 week after injection.

*Agrobacterium rhizogenes* (K599) mediated hairy root transformation was performed on soybean W82 as previously described (Kereszt et al., 2007). In brief, a single colony was picked and grown in 10 ml of liquid LB medium until OD_600_ reached around 0.4 to 0.6. Then the culture was spun down and resuspended in the CCM buffer (MS 0.443 g L^-1^; 3.9 g L^-1^ morpholino ethanesulfonic acid; 150 mg L^-1^ cysteine; and 150 mg L^-1^ dithiothreitol) to reach an OD_600_ of 0.2 to 0.3. Then the roots were incubated in the solution for 2–3 days. The phenotype was observed 4 weeks after transformation.

### Plasmid Construction

The CRISPR/Cas9 vector *pKSE401G* was modified from *pKSE401* (Xing et al., 2014). The vector map was shown in Figure 1. The *35S-sGFP-terminator* cassette was amplified from *pK7GWIWG2D* (Karimi et al., 2007) using primers *35S-GFP-Ter-F* and *35S-GFP-Ter-R*, and inserted into the *PmeI* site of *pKSE401* by the Gibson assembly method (Gibson et al., 2009).

The *GENE-sgRNA* plant expression vectors were constructed as previously described with minor modifications (Xing et al., 2014). The target sgRNA sequences were designed using the web server CRISPR-P^3^ (Lei et al., 2014), and then the sequences were further analyzed by the software CRISPR Primer Designer (Yan M. et al., 2015). Using *pCBC-DT1T2* as the template, two *AtU6 promoter-sgRNA-AtU6 terminator* cassettes were amplified by PCR using the primers listed in Supplementary Table S6. Then the PCR fragments were inserted into *pKSE401G* by Golden Gate Assembly (Gao et al., 2013), and confirmed by Sanger sequencing. These vectors were used for plant transformation.

### Mutant Screening and Validation of Genome Editing

The *Arabidopsis* and *B. napus* mutants were first screened by GFP fluorescence, which was examined using a dissecting microscope equipped with a GFP filter (Olympus, SZX16). Then, the candidates were further confirmed by Cas9 specific primers (Supplementary Table S6).

To analyze the mutations caused by CRISPR/Cas9, genomic DNA was extracted from leaves, fruit, or root using the CTAB method (Molecular Cloning, 3rd edition). The flanking sequences of the CRISPR target sites were amplified by PCR using gene-specific primers (Supplementary Table S6). Then, most of the amplicons were directly sequenced. To decode the mutations, the online tool DSDecode^4^ (Liu et al., 2015) was used for chromatogram decoding. Specifically, the sequence files (xxx.abi) and the reference gene sequences were uploaded to the server and analyzed using default settings. Subsequently, the results were aligned with the reference sequences to ensure that the mutations were in the sgRNA target sites. For complex mutations, the amplicons were first sub-cloned into the *pGEM-T* easy vector (A3600, Promega, United States), and about 10 clones for each amplicon were individually sequenced.

### ABA Treatment and Measurement of Stomatal Aperture

Stomatal assays were performed essentially as previously described (Desikan et al., 2002). Fully expanded leaves (3–4 weeks) were used for this assay. Abaxial epidermal strips from similar rosette leaves were first floated in 10 mM MES buffer (pH 6.15) containing 20 mM KCl for 2.5–3 h under white light (95 E m^-2^ s^-1^) to allow the stomata open. Afterwards, added 1 μM ABA to the buffer and incubated for another 1hr. The stoma images were taken by a ZEISS microscope equipped with a digital camera (AxioCam ICc5, Zeiss). Stomatal aperture was measured using Image J.

### RNA Extraction and qRT-PCR

Total RNA was extracted using a Plant Total RNA Isolation Kit (Sangon Biotech, Shanghai, China, No. SK8631) following the manufacturer’s instructions. Approximately 1 μg of total RNA was used for cDNA synthesis using a PrimeScript^TM^ RT reagent kit (TaKaRa, Japan, Cat#RR047A). For qPCR, a total volume of 10 μl reaction mixture was used containing 5 μl of 2 × SYBR Green master mix (Cat# 172-5124, Bio-Rad), 0.5 μl of 5× diluted cDNA, 0.25 μl of each primer, and 4 μl ddH_2_O. Amplification was performed using a CFX Connect^TM^ system (Bio-Rad, United States). The amplification program consisted of one cycle of 95°C for 5 min, followed by 50 cycles of 95°C for 15s, 60°C for 20 s, and 72°C for 20 s. The fluorescent product was detected at the third step of each cycle. The expression level of each gene was calculated using the 2^-ΔΔCT^ method (Livak and Schmittgen, 2001). All analyses were repeated three times using biological replicates. The *ACTIN* and *GAPDH* genes served as the internal control. All primers are listed in Supplementary Table S6.

### Phylogenetic Analysis

The protein sequences of genes in *Arabidopsis thaliana, B. napus*, *B. rapa*, and *B. oleracea* were obtained from the website http://planttfdb.cbi.pku.edu.cn/index.php (Jin et al., 2017). The sequence alignment was performed using Clustal Omega^5^. An unrooted phylogenetic tree was constructed using MEGA7^6^(Kumar et al., 2016) with the neighbor-joining statistical method and bootstrap analysis (1000 replicates).

## Author Contributions

CD and TT designed the research. TT, XY, HY, QG, HJ, YW, GY, HL, YP, KL, XL, CM, and CD performed the experiments. TT, XY, HJ, CM, and CD analyzed the data. CD, CK, TT, and CM wrote the manuscript. All authors read and approved the manuscript.

## Conflict of Interest Statement

The authors declare that the research was conducted in the absence of any commercial or financial relationships that could be construed as a potential conflict of interest.

## Footnotes

1 http://dsdecode.scgene.com/

2 http://cbi.hzau.edu.cn/cgi-bin/CRISPR

3 http://cbi.hzau.edu.cn/cgi-bin/CRISPR

4 http://dsdecode.scgene.com/

5 http://www.ebi.ac.uk/Tools/msa/clustalo

6 http://www.megasoftware.net/
