# Supplementary material for: Development and Validation of an Effective CRISPR/Cas9 Vector for Efficiently Isolating Positive Transformants and Transgene-Free Mutants in a Wide Range of Plant Species
Source: Front Plant Sci. 2018 Oct 23;9:1533. doi: 10.3389/fpls.2018.01533 (PMC6206294; doi:10.3389/fpls.2018.01533)
Supplement: Supplementary file 1 [file Data_Sheet_1.docx]

**Supplemental Information**

**Figure S1.** Gel image showing the presence of *Cas9* in *CR-rpk1* (T1) and *CR-bnaarf2* (T0).

**Figure S2.** Gel image showing the presence of *GFP*, *Cas9*, *NPTII* and *U6-26* in different progeny of *CR-rpk1* and *CR-bnaarf2*.

**Figure S3.** Genotypes of the *CR-bnaarf2* mutants.

**Figure S4.** Expression of *RPK1* and *BnaARF2s* in the CRISPR/Cas9 mutants obtained by qRT-PCR.

**Figure S5.** Phylogenetic analysis of the ARF2 homologs in *Arabidopsis* and *B. napus*, *B. rapa*, and *B. oleracea*.

**Figure S6.** Genotypes of strawberry fruit transiently transformed with *CR-FveMyb10*.

**Figure S7.** Number of nodules induced in soybean roots transiently transformed with *CR-GmNFR1a*.

**Figure S8.** Genotypes of soybean roots transiently transformed with *CR-GmNFR1a*.

**Table S1.** Percentage of *CR-rpk1* (T2) or *CR-banarf2* (T1) with or without *Cas9*.

**Table S2.** Segregation patterns of Cas9-free mutants in the T2 generation in *Arabidopsis*.

**Table S3.** Segregation patterns of Cas9-free mutants in the T3 generation in *Arabidopsis*.

**Table S4.** Segregation patterns of Cas9-free mutants in the T1 generation in *B. napus*.

**Table S5.** Segregation patterns of Cas9-free mutants in the T2 generation in *B. napus*.

**Table S6.** The list of primers used in this study.

**Table S7.** Summary of the sgRNAs sequence (%GC contents and mutation rates). The PAM motif (NGG) is marked by a grey box.

**
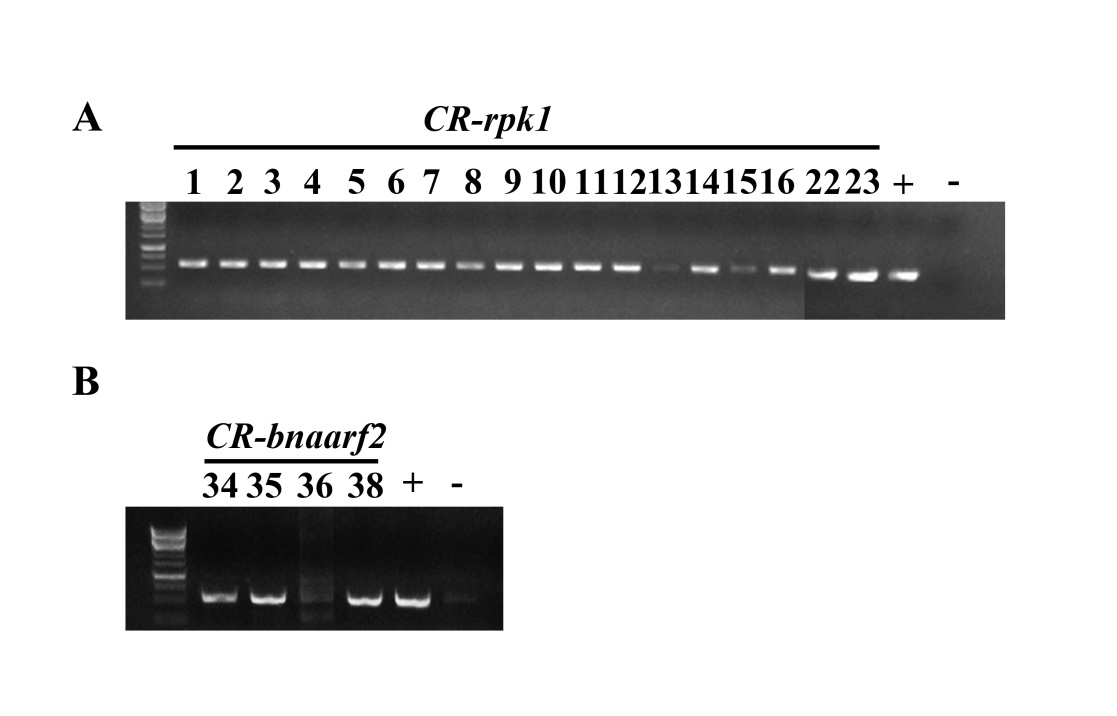
**

**Figure S1.** Gel image showing the presence of *Cas9* in *CR-rpk1* (T1) and *CR-bnaarf2* (T0).

(A) The Cas9 fragment was amplified by Cas9 primers in *CR- rpk1* (L1-L23; T1 generation). (B) The Cas9 fragment was amplified by Cas9 primers in *CR-bnaarf2* (L34, L35, L36 and L38). +: The vector pKSE401 was used as the positive control; -: gDNA of WT was used as the negative control.

**
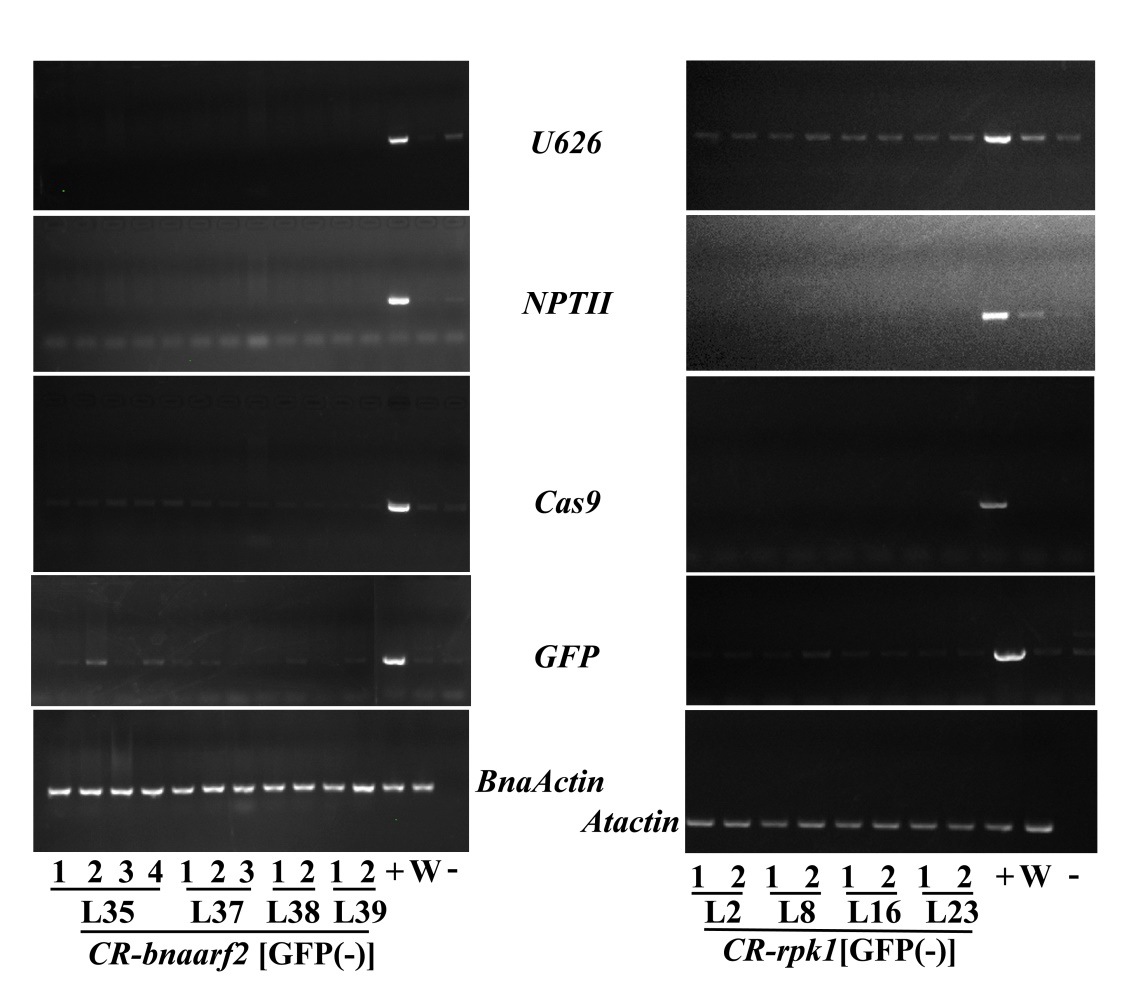
**

**Figure S2.** Gel image showing the presence of *GFP*, *Cas9*, *NPTII* and *U6-26* in different progeny of *CR-rpk1* and *CR-bnaarf2*.

+: pKSE401 was used as the positive control for *Cas9, NPTII and U626*. -: gDNA from *Westar* or *Col* were used as the negative control for *Cas9, NPTII and U626*. ddH_2_O was used as the negative control for *BnaActin1* or *Atactin*. The *B. napus* or *Arabidopsis* actin genes were used as the PCR internal control.

**
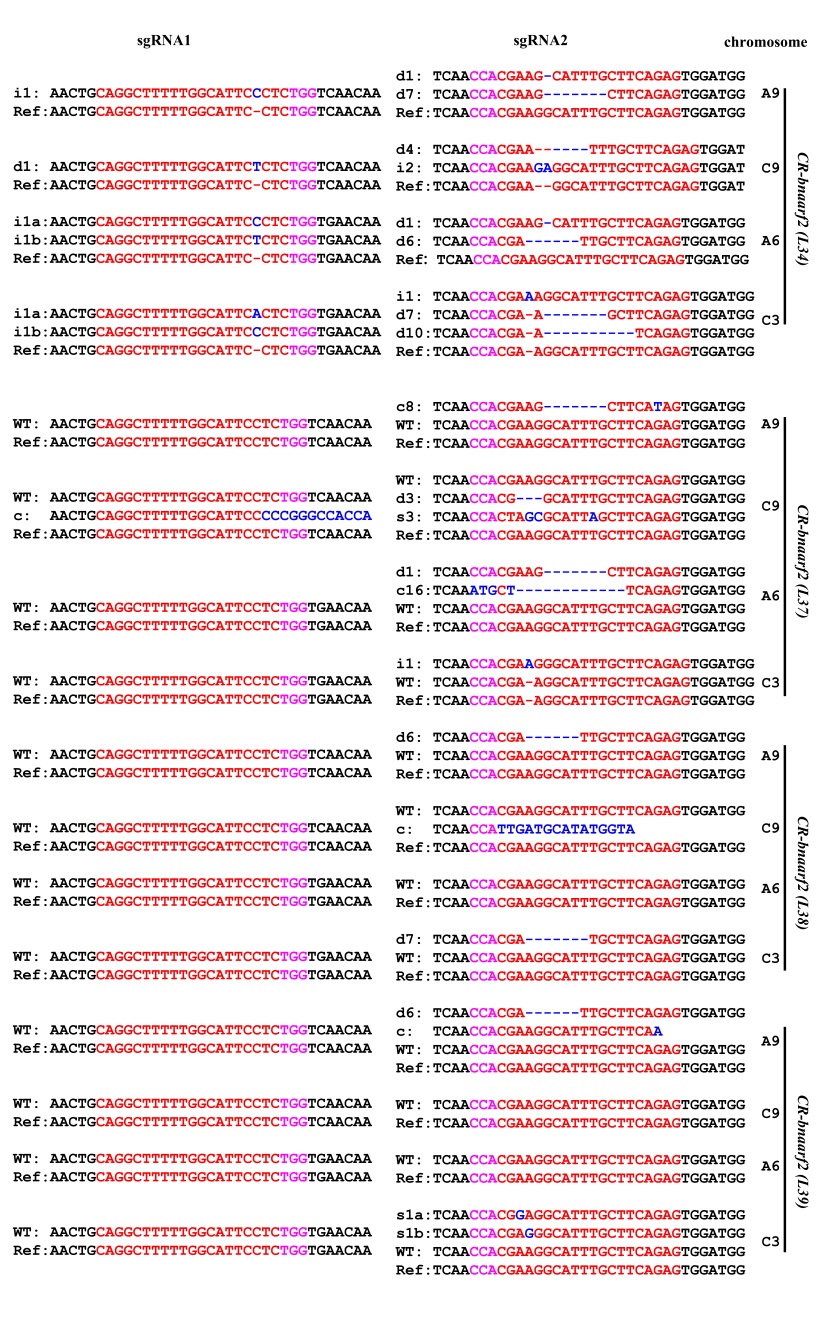
**

**Figure S3.** Genotypes of the *CR-bnaarf2* mutants.

The PAM sequence is indicated with magenta. The sgRNA is indicated with red. The mutation sites are indicated with blue. i: insertion; d: deletion; c: combined. d#, # of base pair (bp) deleted from target site; i#, # of bp inserted at target site, c#, combined mutations. L34, L37, L38 and L39 indicated individual T0 transgenic plants.

**
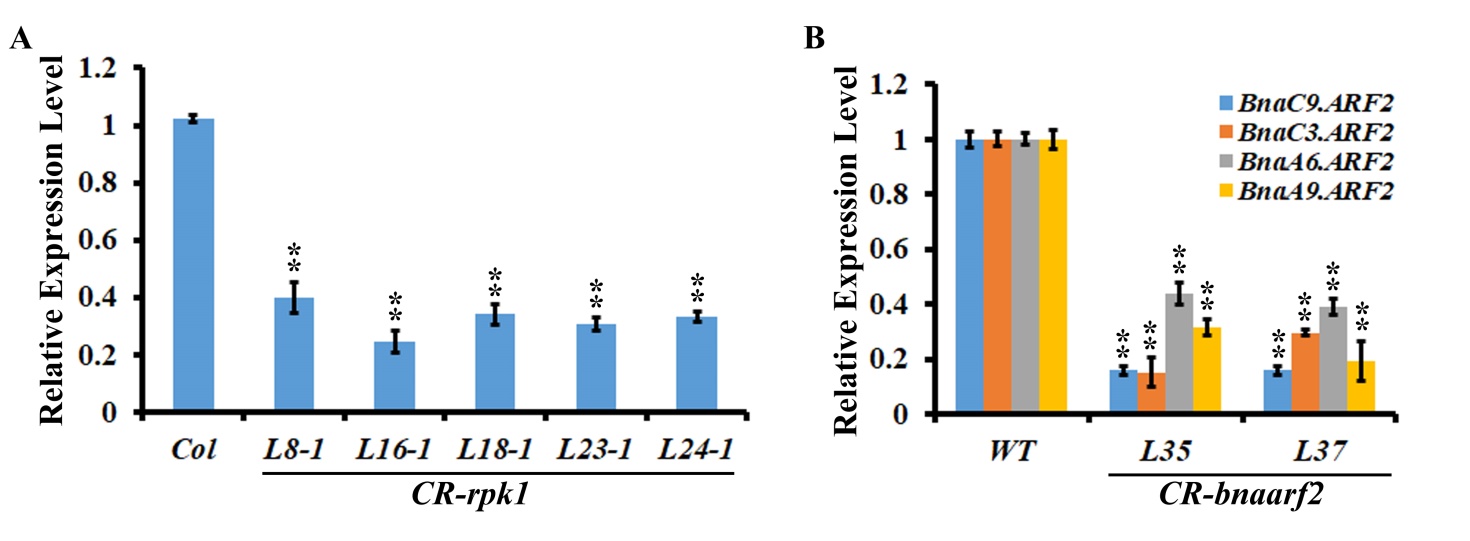
**

**Figure S4.** Expression of *RPK1* and *BnaARF2s* in the CRISPR/Cas9 mutants obtained by qRT-PCR.

(A) qRT-PCR analysis revealed the suppressed expression of *RPK1* in *CR-rpk1* mutant plants. The *Arabidopsis* *GAPDH* gene was amplified and used as an internal control. (B) qRT-PCR analysis revealed the suppressed expression of *BnaARF2* paralogues in *CR-bnaarf2* quadruple mutant plants (L35 and L37). The *B.napus* *GAPDH* gene was amplified and used as an internal control. In A and B, data are means ± SD obtained from three biological replicates; **, P < 0.01, Student’s *t*-test.

**
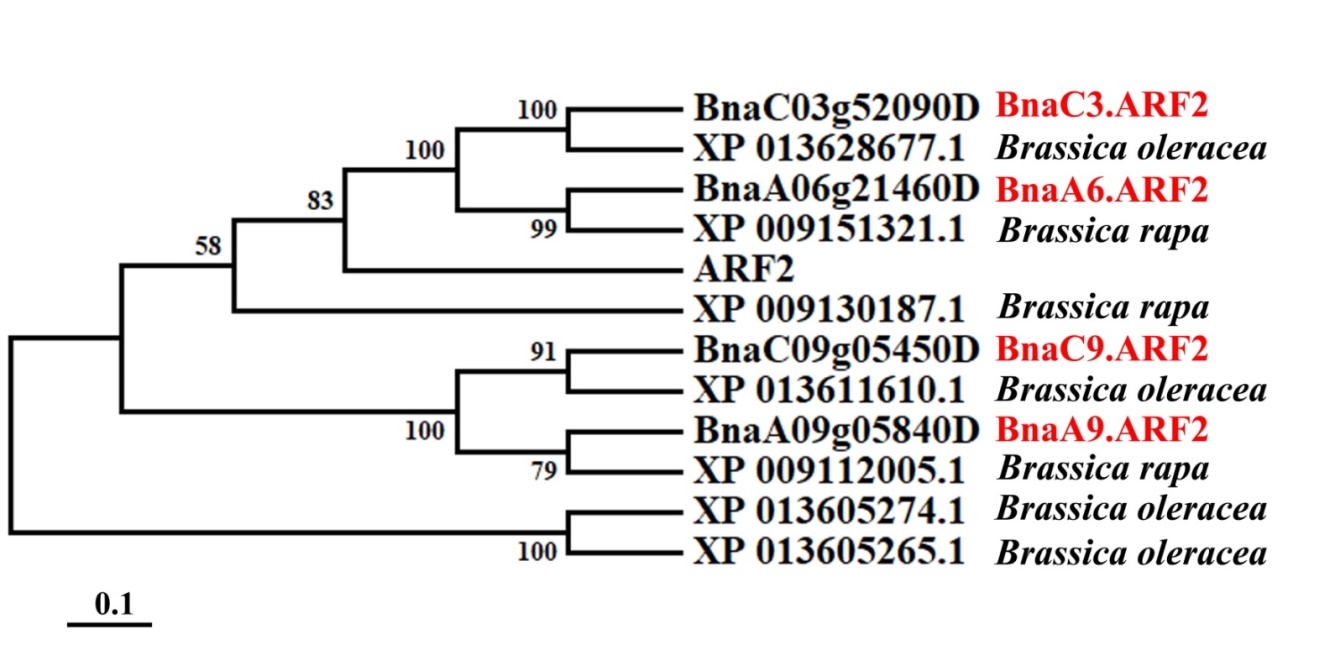
**

**Figure S5.** Phylogenetic analysis of the ARF2 homologs in *Arabidopsis* and *B. napus*, *B. rapa*, and *B. oleracea*.

**
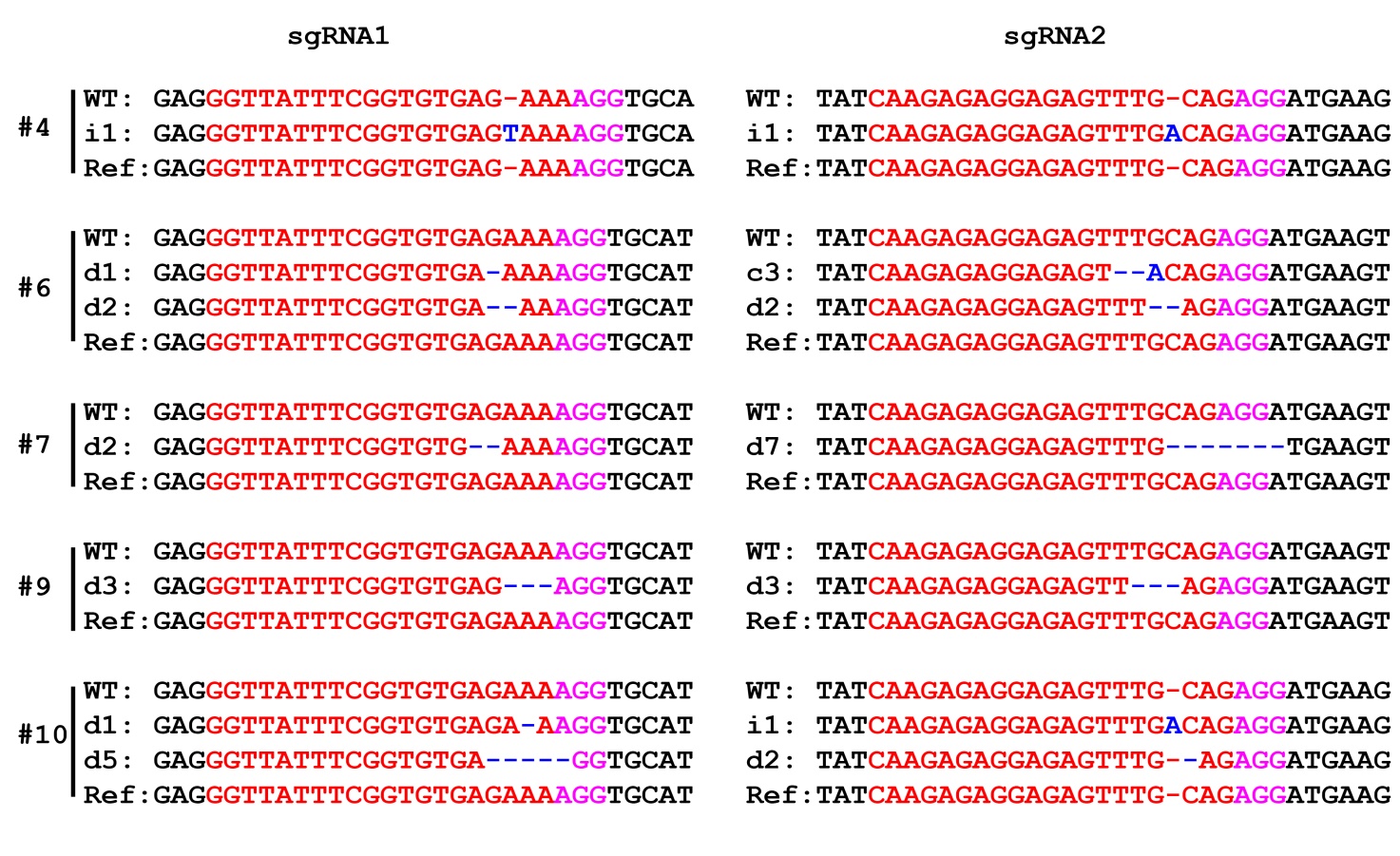
**

**Figure S6.** Genotypes of strawberry fruit transiently transformed with *CR-FveMyb10*.

The PAM sequence is indicated with magenta. The sgRNA is indicated with red. The mutation sites are indicated with blue. i: insertion; d: deletion; c: combined. d#, # of base pair (bp) deleted from target site; i#, # of bp inserted at target site, c#, combined mutations. #4, #6, #7, #9, and #10 indicated individual transgenic fruit.

**
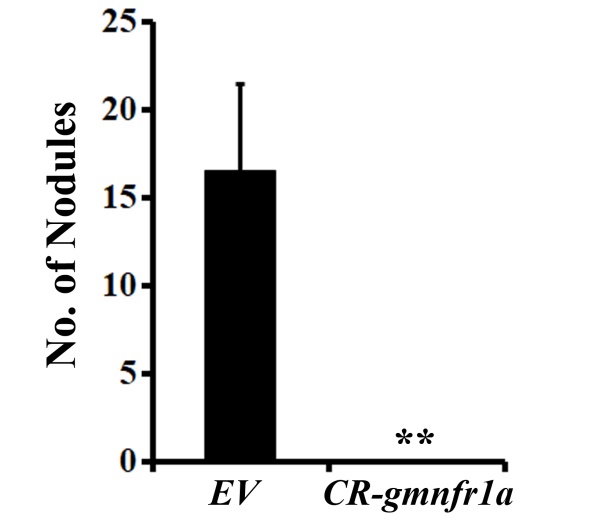
**

**Figure S7.** Number of nodules induced in soybean roots transiently transformed with *CR-GmNFR1a*.

Bar graph shows the nodule number of mutated roots induced by *CR-gmnfr1a* and control roots four weeks after infection. Error bars represent SD (n=6). **, P < 0.01, Student’s *t*-test.

**
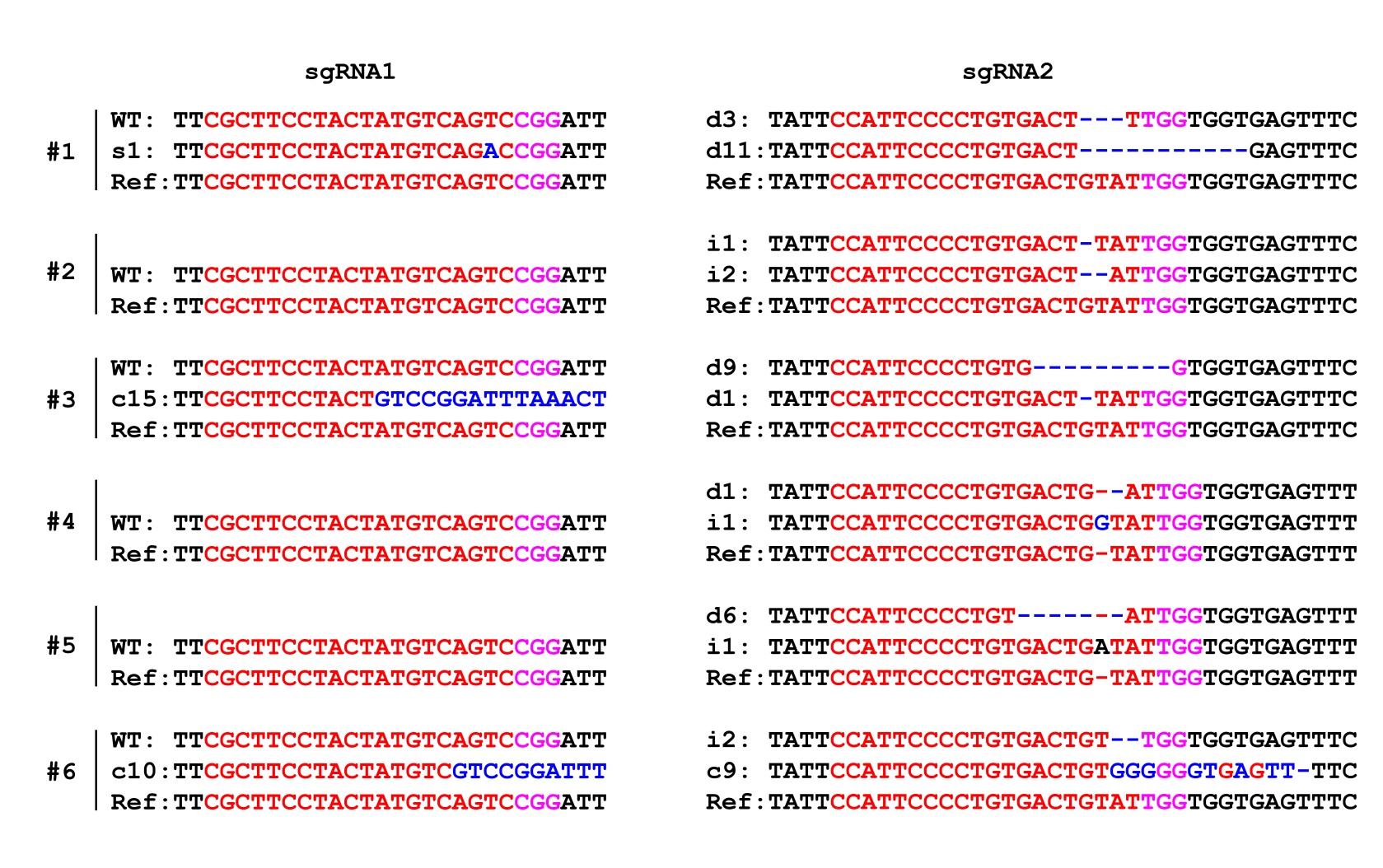
**

**Figure S8.** Genotypes of soybean roots transiently transformed with *CR-GmNFR1a*.

The PAM sequence is indicated with magenta. The sgRNA is indicated with red. The mutation sites are indicated with blue. i: insertion; d: deletion; c: combined. d#, # of base pair (bp) deleted from target site; i#, # of bp inserted at target site, c#, combined mutations. #1 to #6 indicated individual transgenic root.

**Table S1.** Percentage of *CR-rpk1* (T2) or *CR-banarf2* (T1) with or without *Cas9*.

| **Target Gene** | **Line** | **No. of plants examined** | **GFP positive plants** | **GFP positive rate** | **GFP negative plants** | **GFP negative rate** | **Cas9 negative plants (total examined plant)** | **Cas9 negative rate** |
| --- | --- | --- | --- | --- | --- | --- | --- | --- |
| ***RPK1* (T2)** | **L2** | **25** | **18** | **72%** | **7** | **28%** | **6 (6)** | **100.0%** |
|  | **L6** | **14** | **14** | **100%** | **0** | **0** |  |  |
|  | **L6** | **12** | **9** | **75%** | **3** | **25%** | **3 (3)** | **100.0%** |
|  | **L7** | **10** | **7** | **70%** | **3** | **30%** | **3 (3)** | **100.0%** |
|  | **L8** | **47** | **34** | **72.34%** | **13** | **27.66%** | **9 (9)** | **100.0%** |
|  | **L16** | **19** | **19** | **100%** | **0** | **0** |  |  |
|  | **L17** | **33** | **24** | **72.73%** | **9** | **27.27%** | **8 (8)** | **100.0%** |
|  | **L18** | **29** | **28** | **96.56%** | **1** | **3.44%** | **8 (8)** | **100.0%** |
|  | **L23** | **62** | **53** | **85.48%** | **9** | **14.52%** | **0 (1)** | **0** |
| **Total (average)** |  | **251** | **206** | **(82.1%)** | **45** | **(17.3%)** | **37** | **(85.7%)** |
| ***BnaARF2* (T1)** | **L35** | **32** | **22** | **68.75%** | **10** | **31.25%** | **10 (10)** | **100.0%** |
|  | **L37** | **34** | **27** | **79.41%** | **7** | **20.59%** | **7 (7)** | **100.0%** |
|  | **L38** | **17** | **14** | **82.35%** | **3** | **17.65%** | **3(3)** | **100.0%** |
|  | **L39** | **12** | **12** | **66.67%** | **4** | **33.33%** | **4 (4)** | **100.0%** |
| **Total (average)** |  | **95** | **71** | **(74.7%)** | **24** | **(25.7%)** | **24** | **(100.0%)** |

**Table S2: Segregation patterns of Cas9-free mutants in the *Arabidopsis* T2 generation.**

|  | | | T1 | | |  | T2 | |  |
| --- | --- | --- | --- | --- | --- | --- | --- | --- | --- |
| Target gene | **sgRNA** | **Line** | **Zygosity** ◐ | **Genotype** | **Cas9**§ |  | **Genotype** | **Cas9**§ | |
| *RPK1* | **sgRNA1** | **L2** | **Bi-allelic** | **i1a:i1b** | **Yes** |  | **4i1ai1a:2i1ai1b** | **No** | |
| *RPK1* | **sgRNA1** | **L6** | **Heterozygous** | **i1:WT** | **Yes** |  | **3i1WT** | **No** | |
| *RPK1* | **sgRNA1** | **L7** | **Heterozygous** | **i1:WT** | **Yes** |  | **2i1i1:1i1WT** | **No** | |
| *RPK1* | **sgRNA1** | **L8** | **Bi-allelic** | **i1a:i1b** | **Yes** |  | **3i1ai1a:3i1ai1b:3i1bi1b** | **No** | |
| *RPK1* | **sgRNA1** | **L17** | **Bi-allelic** | **i1a:i1b** | **Yes** |  | **5i1ai1a:3i1ai1b** | **No** | |
| *RPK1* | **sgRNA1** | **L18** | **Heterozygous** | **i1:WT** | **Yes** |  | **4i1i1:2i1WT:2WTWT** | **No** | |

i#, # of bp inserted at target site; i#a, same number of insertion at one site; i#b, same number of insertion of different nucleotide at the same site.

◐: the zygosity of homozygote, bi-allele, and heterozygote in T1and T2 plants. §: presence of Cas9 sequence: Yes, Cas9 positive; No, Cas9 negative.

**Table S3.** Segregation patterns of Cas9-free mutants in the T3 generation in *Arabidopsis*.

|  | | | T2 | | |  | T3 | |  |
| --- | --- | --- | --- | --- | --- | --- | --- | --- | --- |
| Target gene | **sgRNA** | **Line** | **Zygosity** ◐ | **Genotype** | **Cas9**§ |  | **Genotype** | **Cas9**§ | |
| *RPK1* | **sgRNA1** | **L8-3** | **Homozygous** | **i1i1** | **No** |  | **10i1i1** | **No** | |
| *RPK1* | **sgRNA1** | **L18-1** | **Homozygous** | **i1i1** | **No** |  | **10i1i1** | **No** | |

d#b, same number of deletion at other sites; i#, # of bp inserted at target site.

◐: the zygosity of homozygote, bi-allele, and heterozygote in T2 and T3 plants. §: presence of Cas9 sequence: Yes, Cas9 positive; No, Cas9 negative.

**Table S4.** Segregation patterns of Cas9-free mutants in the T1 generation in *B. napus*.

|  | | | T0 | | |  | T1 | |  |
| --- | --- | --- | --- | --- | --- | --- | --- | --- | --- |
| Target gene | **sgRNA** | **Line** | **Zygosity** ◐ | **Genotype** | **Cas9**§ |  | **Genotype** | **Cas9**§ | |
| BnaA9.ARF2 | **sgRNA2** | **L35** | **Chimera** | **d9:d10:s3** | **Yes** |  | **5d9d10:5h** | **No** | |
| BnaC9.ARF2 | **sgRNA2** | **L35** | **Bi-allelic** | **d8:s1** | **Yes** |  | **3d8d8:4d8s1:3s1s1** | **No** | |
| BnaA6.ARF2 | **sgRNA2** | **L35** | **Heterozygous** | **i1:WT** | **Yes** |  | **4i1i1:5i1WT:1WTWT** | **No** | |
| BnaC3.ARF2 | **sgRNA2** | **L35** | **Heterozygous** | **d1:WT** | **Yes** |  | **3d2d2:4d2WT:3WTWT** | **No** | |
| BnaC3.ARF2 | **sgRNA2** | **L37** | **Heterozygous** | **i1:WT** | **Yes** |  | **5i1i1:2i1WT** | **No** | |
| BnaC9.ARF2 | **sgRNA2** | **L37** | **Chimera** | **d3:s3:WT** | **Yes** |  | **7h** | **No** | |
| BnaA9.ARF2 | **sgRNA2** | **L38** | **Heterozygous** | **d6:WT** | **Yes** |  | **3d6WT** | **No** | |
| BnaC3.ARF2 | **sgRNA2** | **L38** | **Heterozygous** | **d7:WT** | **Yes** |  | **3d7WT** | **No** | |

d#, # of bp deleted from target site; i#, # of bp inserted at target site; s#, # of bp substituted of the target site; h, heterogeneous, more than one sequence detected in the sample.

◐: the zygosity of homozygote, bi-allele, and heterozygote in T0 and T1 plants. §: presence of Cas9 sequence: Yes, Cas9 positive; No, Cas9 negative.

**Table S5.** Segregation patterns of Cas9-free mutants in the T2 generation in *B. napus*.

|  | | | T1 | | |  | T2 | |  |
| --- | --- | --- | --- | --- | --- | --- | --- | --- | --- |
| Target gene | **sgRNA** | **Line** | **Zygosity** ◐ | **Genotype** | **Cas9**§ |  | **Genotype** | **Cas9**§ | |
| BnaA9.ARF2 | **sgRNA2** | **L35-1** | **Bi-allelic** | **d9:d10** | **No** |  | **3d9d9:5d9d10:2d10d10** | **No** | |
| BnaC9.ARF2 | **sgRNA2** | **L35-1** | **Homozygous** | **d8d8** | **No** |  | **10d8d8** | **No** | |
| BnaA6.ARF2 | **sgRNA2** | **L35-1** | **Homozygous** | **d1d1** | **No** |  | **10d1d1** | **No** | |
| BnaC3.ARF2 | **sgRNA2** | **L35-1** | **Homozygous** | **d2d2** | **No** |  | **10d2d2** | **No** | |

d#, # of bp deleted from target site.

◐: the zygosity of homozygote, bi-allele, and heterozygote in T1 and T2 plants. §: presence of Cas9 sequence: Yes, Cas9 positive; No, Cas9 negative.

**Table S6.** The list of primers used in this study.

| **Primer name** | **Primer sequence (5’ to 3’)** | **Purpose of the primers** |
| --- | --- | --- |
| RPK1-DT1-BsF | ATATATGGTCTCGATTGCTAACTGTATCGGTCACGGGTT | Making RPK1 CRISPR construct |
| RPK1-DT1-F0 | TGCTAACTGTATCGGTCACGGGTTTTAGAGCTAGAAATAGC | Making RPK1 CRISPR construct |
| RPK1-DT2-R0 | AACAAGGAGATTTGGGGGTTGGCAATCTCTTAGTCGACTCTAC | Making RPK1 CRISPR construct |
| RPK1-DT2-BsR | ATTATTGGTCTCGAAACAAGGAGATTTGGGGGTTGGCAA | Making RPK1 CRISPR construct |
| BnaARF2-DT1-BsF | ATATATGGTCTCGATTGAGGCTTTTTGGCATTCCTCGTT | Making BnaARF2 CRISPR construct |
| BnaARF2-DT1-F0 | TGAGGCTTTTTGGCATTCCTCGTTTTAGAGCTAGAAATAGC | Making BnaARF2 CRISPR construct |
| BnaARF2-DT2-R0 | AACCGAAGGCATTTGCTTCAGACAATCTCTTAGTCGACTCTAC | Making BnaARF2 CRISPR construct |
| BnaARF2-DT2-BsR | ATTATTGGTCTCGAAACCGAAGGCATTTGCTTCAGACAA | Making BnaARF2 CRISPR construct |
| FvMyb10-DT1-BsF | ATATATGGTCTCGATTGGTTATTTCGGTGTGAGAAAGTT | Making FvMyb10 CRISPR construct |
| FvMyb10-DT1-F0 | TGGTTATTTCGGTGTGAGAAAGTTTTAGAGCTAGAAATAGC | Making FvMyb10 CRISPR construct |
| FvMyb10-DT2-R0 | AACCTGCAAACTCTCCTCTCTTCAATCTCTTAGTCGACTCTAC | Making FvMyb10 CRISPR construct |
| FvMyb10-DT2-BsR | ATTATTGGTCTCGAAACCTGCAAACTCTCCTCTCTTCAA | Making FvMyb10 CRISPR construct |
| GmNFR1a-DT1-BsF | ATATATGGTCTCGATTGGCTTCCTACTATGTCAGTCGTT | Making GmNFR1a CRISPR construct |
| GmNFR1a -DT1-F0 | TGGCTTCCTACTATGTCAGTCGTTTTAGAGCTAGAAATAGC | Making GmNFR1a CRISPR construct |
| GmNFR1a -DT2-R0 | AACATACAGTCACAGGGGAATGCAATCTCTTAGTCGACTCTAC | Making GmNFR1a CRISPR construct |
| GmNFR1a -DT2-BsR | ATTATTGGTCTCGAAACATACAGTCACAGGGGAATGCAA | Making GmNFR1a CRISPR construct |
| GmNFR1a–checking-F | ACCCTGTATAGAAAGTCCTAG | Checking GmNFR1a gene editing |
| GmNFR1a–checking-R | CATTAACCCTGGCATTTGCAG | Checking GmNFR1a gene editing |
| AtRPK1-DS1_sgR1-F | CTAACTGTATCGGTCACGG | Checking RPK1-sgRNA1 gene editing |
| AtRPK1-DS1_sgR1-R | TCTCGGTTTCGCTCGCATGGTAAC | Checking RPK1-sgRNA1 gene editing |
| AtRPK1-DS2_sgR2-F | CGAGAGTGGTGGTGGTCTCCACG | Checking RPK1-sgRNA2 gene editing |
| AtRPK1-DS2_sgR2-R | AACCCCCAAATCTCCTTTGG | Checking RPK1-sgRNA2 gene editing |
| FvMyb10-checking-F | TTCTCTTTCGTAAAGTAATTAATG | Checking FvMyb10 gene editing |
| FvMyb10-checking-R | TAGCTTTGGAATTCCAGAAG | Checking FvMyb10gene editing |
| BnaC9.ARF2-sgRNA1-F | CTTCTAACCCATTTTCCTGAGTTTTGG | Checking BnaC9.ARF2-sgRNA1 gene editing |
| BnaC3.ARF2-sgRNA1-F | CTGTTTTTGTTAGATCATACTATTATTCAAGAAGCC | Checking BnaC3.ARF2-sgRNA1 gene editing |
| BnaA9.ARF2-sgRNA1-F | CACTTGCTTCTTATTTGTATTGAAACATATTTGAC | Checking BnaA9.ARF2-sgRNA1 gene editing |
| BnaA6.ARF2-sgRNA1-F | GCAGAGTATAACAGGTTGATCATATTATTCAAG | Checking BnaA6.ARF2-sgRNA1 gene editing |
| BnaARF2-sgRNA1-R | GCCTTACACCCACACGTAATTC | Conserved primer for BnaARF2-sgRNA1 site |
| BnaARF2-sgRNA2-F | AACTGGCCAATACGTCCAC | Conserved primer for BnaARF2-sgRNA2 site |
| BnaC9.ARF2-sgRNA2-R | AAGTTATTAATTCAGAAGAACACACAAGTATAA | Checking BnaC9.ARF2-sgRNA2 gene editing |
| BnaC3.ARF2-sgRNA2-R | CTCTGCGTTACAAAAGTCTCGTTAGT | Checking BnaC3.ARF2-sgRNA2 gene editing |
| BnaA9.ARF2-sgRNA2-R | CTCTCTAGTAACCTGTGAACACATATGTA | Checking BnaA9.ARF2-sgRNA2 gene editing |
| **Primer name** | **Primer sequence (5’ to 3’)** | **Purpose of the primers** |
| BnaA6.ARF2-sgRNA2-R | AACACCCAAGTCTCATTTGTCTGTAAT | Checking BnaA6.ARF2-sgRNA2 gene editing |
| 35S-GFP-Ter-F | CTGTCAAACACTGATAGTTTTGCATGCCTGCAGGTCAAC | Amplify 35S-GFP-Terminator cassette |
| 35S-GFP-Ter-R | TCGTTTCCCGCCTTCAGTTTTCGACCATATGGGAGAGCTC | Amplify 35S-GFP-Terminator cassette |
| Cas9-s | AGACCGTGAAGGTTGTGGAC | Cas9 gene checking primer |
| Cas9-r | TAGTGATCTGCCGTGTCTCG | Cas9 gene checking primer |
| NPTII-s | ATGGGGATTGAACAAGATGGAT | NPTII gene checking primer |
| NPTII-r | CAGAAGAACTCGTCAAGAAGGCG | NPTII gene checking primer |
| U626-s | TGTCCCAGGATTAGAATGATTAGGC | pKSE401 fragment checking primer |
| U629-r | GTCAGGCTGCAGTAGTTTCCATTAA | pKSE401 fragment checking primer |
| GFP-s | ATGGTGAGCAAGGGCGAGGAGC | GFP gene checking primer |
| GFP-r | TTACTTGTACAGCTCGTCCATGC | GFP gene checking primer |
| BnaActin-s | ATGTGATGTGGATATCAGGAAGGAT | qPCR primer of *B.napus* internal control gene |
| BnaActin-r | ACGGTCCAGATTCGTCATACTCA | qPCR primer of *B.napus* internal control gene |
| Atactin2-s | GTTCCAGCCCTCGTTTGTG | qPCR primer of *Arabidopsis* internal control gene |
| Atactin2-r | CAAGTGCTGTGATTTCTTTGCTC | qPCR primer of *Arabidopsis* internal control gene |
| RPK1-sgRNA2-off--F1 | GAAAAGTTGGAAGTAACTTATCCG | Checking RPK1 sgRNA2 off-targeted site |
| RPK1-sgRNA2-off-R1 | ACCTTCAGTTTGTATATCATTATTCATT | Checking RPK1 sgRNA2 off-targeted site |
| RPK1-sgRNA2-off-F2 | CAAGAAATAGATAAACTGTGTTCAAG | Checking RPK1 sgRNA2 off-targeted site |
| RPK1-sgRNA2-off-R2 | GACAGCCTATATTACTCCATTCATC | Checking RPK1 sgRNA2 off-targeted site |
| ARF2-sgRNA1-off-F1 | ATCTATGGTATTGGCAGGATAACG | Checking BnaARF2 sgRNA1 off-targeted site1 |
| ARF2-sgRNA1-off-R1 | GTTAAAGCTTCATTCTCATAATATTTCATC | Checking BnaARF2 sgRNA1 off-targeted site1 |
| ARF2-sgRNA1-off-F2 | GATCTTCCAAAAAGGTATTAAACAACGGAAGAG | Checking BnaARF2 sgRNA1 off-targeted site2 |
| ARF2-sgRNA1-off-R2 | CTCACCGTAGTGTCATTCCAAGAGAATG | Checking BnaARF2 sgRNA1 off-targeted site2 |
| ARF2-sgRNA1-off-F3 | GGTTTTACACACACTAAGACTCGCCAC | Checking BnaARF2 sgRNA1 off-targeted site3 |
| ARF2-sgRNA1-off-R3 | TAAAGCTCGAGCACAGCTTCCAAATC | Checking BnaARF2 sgRNA1 off-targeted site3 |
| ARF2-sgRNA2-off-F1 | AGGATATGTCACGTCAGCCTCCT | Checking BnaARF2 sgRNA2 off-targeted site1 |
| ARF2-sgRNA2-off-R1 | TAATTCTCCATTCTCACCCCTACGCAA | Checking BnaARF2 sgRNA2 off-targeted site1 |
| ARF2-sgRNA2-off-F2 | CAGGATCTCCGGTCAATCTCCCTA | Checking BnaARF2 sgRNA2 off-targeted site2 |
| ARF2-sgRNA2-off-R2 | CAAAAATATCAAGCTCCTCCCCTGTTCATG | Checking BnaARF2 sgRNA2 off-targeted site2 |
| ARF2-sgRNA2-off-F3 | CCTGGGATTCCGTGGCATTACTTT | Checking BnaARF2 sgRNA2 off-targeted site3 |
| ARF2-sgRNA2-off-R3 | AAACTGGAGATACTCAGCAGAGGGATC | Checking BnaARF2 sgRNA2 off-targeted site3 |
| ARF2-A6-qPCR-R | CTACTATCCTCGGACTAGGCGA | qT-PCR primer for BnaA6.ARF2 |
| ARF2-A6-qPCR-F | TATGGTTCATACGTACAAATGCTGC | qT-PCR primer for BnaA6.ARF2 |
| ARF2-A9-qPCR-R | AGATTCATGTAACGTCAGGGAAAGGC | qT-PCR primer for BnaA9.ARF2 |
| **Primer name** | **Primer sequence (5’ to 3’)** | **Purpose of the primers** |
| ARF2-A9-qPCR-F | TGATGTGGTTTCGGCTTCAGCTTC | qT-PCR primer for BnaA9.ARF2 |
| ARF2-C3-qPCR-F | TTTGAGCTCTCTCGCATAGTAG | qT-PCR primer for BnaC3.ARF2 |
| ARF2-C3-qPCR-R | TGGTTCATTTTAGGGTGCTAGT | qT-PCR primer for BnaC3.ARF2 |
| ARF2-C9-qPCR-F | CAGACATGAATCTGCTTACACGGA | qT-PCR primer for BnaC9.ARF2 |
| ARF2-C9-qPCR-R | CGGCCACAGAAGGTAGTGATG | qT-PCR primer for BnaC9.ARF2 |
| ATRPK1-qPCR-F | CTCTTGGACAACAACTACAACG | qT-PCR primer for RPK1 |
| ATRPK1-qPCR-R | TACTCTGGAGCTACATATCCGA | qT-PCR primer for RPK1 |
| GAPDH-s | TTTGCATCAGAAGGACCACTTAGGG | qPCR primer of *Arabidopsis* internal control gene |
| GAPDH-r | CGTTGGCGGGATATGGTTTTAGC | qPCR primer of *Arabidopsis* internal control gene |
| BnaGAPDH-s | TGCTAAGGTGGTGCATGAGG | qPCR primer of *B.napus* internal control gene |
| BnaGAPDH-r | GTGTTGGAACACGGAAAGCC | qPCR primer of *B.napus* internal control gene |

**Table S7.** Summary of the sgRNAs sequence (%GC contents and mutation rates). The PAM motif (NGG) is marked by a grey box.

| **Target genes** | **Target sequence (PAM in gray)** | **GC content** | **Mutation rate** |
| --- | --- | --- | --- |
| ***RPK1*** | **sgRNA1: CTAACTGTATCGGTCACGGCGG** | **50%** | **33.3%** |
|  | **sgRNA2: TCCAACCCCCAAATCTCCTTTGG** | **50%** | **7.4%** |
| ***FveMyb10*** | **sgRNA1: GGTTATTTCGGTGTGAGAAAAGG** | **40%** | **80%** |
|  | **sgRNA2: CAAGAGAGGAGAGTTTGCAGAGG** | **50%** | **100%** |
| ***GmNFR*** | **sgRNA1: CGCTTCCTACTATGTCAGTCCGG** | **50%** | **50%** |
|  | **sgRNA2: CCATTCCCCTGTGACTGTATTGG** | **50%** | **100%** |
| ***BnaARF2*** | **sgRNA1: CAGGCTTTTTGGCATTCCTCTGG** | **50%** | **BnaA6.ARF2:20%; BnaA9.ARF2:20%; BnaC3.ARF2:20%; BnaC9.ARF2:20%** |
|  | **sgRNA2: CTCTGAAGCAAATGCCTTCGTGG** | **50%** | **BnaA6.ARF2:60%; BnaA9.ARF2:100%; BnaC3.ARF2:100%; BnaC9.ARF2:80%** |
